# Hidden in Plain Sight: Pancreatic and Gastric Metastases of Renal Cell Carcinoma Undetectable on Imaging

**DOI:** 10.7759/cureus.70211

**Published:** 2024-09-25

**Authors:** Madison B Peregoy, Charleston R Powell, Lawrence Goldkind, Dean Baird, Jeffrey A Sanford

**Affiliations:** 1 Internal Medicine, Walter Reed National Military Medical Center, Bethesda, USA; 2 Gastroenterology, Walter Reed National Military Medical Center, Bethesda, USA; 3 Radiology, Walter Reed National Military Medical Center, Bethesda, USA; 4 Pathology, Walter Reed National Military Medical Center, Bethesda, USA

**Keywords:** metastatic renal cell, obstructive jaundice, pancreatic metastasis, radiographic imaging, stomach metastasis

## Abstract

Renal cell carcinoma (RCC) is one of the most common origins of pancreatic metastases. Pancreatic spread of RCC is often found incidentally on surveillance imaging in asymptomatic patients. We present the case of a 49-year-old male with a history of RCC who presented with acute pancreatitis. Multiple imaging studies showed no pancreatic masses, but endoscopic ultrasound with fine needle biopsy and endoscopy showed RCC metastases to the pancreas and stomach, respectively. Pancreatic metastases should be suspected in patients with RCC who present with progressive abdominal pain and biliary obstruction despite negative imaging. Endoscopy is useful for their detection.

## Introduction

Pancreatic metastases from all primary malignancies are rare but well-reported in renal cell carcinoma (RCC), lung, breast, colorectal, and melanomatous cancers [1]. Hypotheses for the proclivity of RCC to metastasize to the pancreas include lymphatic spread and tissue compatibility of the pancreas which allows the proliferation of RCC [2]. The presentation of a pancreatic metastasis from RCC is usually radiographically evident, yet clinically silent [3]. Isolated pancreatic metastases are frequently found on surveillance computed tomography (CT) or when obtained for a different indication. When presenting clinically, symptoms associated with RCC metastases to the pancreas may reflect obstruction from the mass with epigastric pain or painless jaundice. There can be less specific upper gastrointestinal symptoms such as early satiety [3,4].

RCC metastases in the pancreas appear hypervascular and show homogenous contrast enhancement in the arterial phase on CT [2,4,5]. Most cases are seen on CT and magnetic resonance imaging (MRI) but endoscopic ultrasonography (EUS) is more sensitive in assessing pancreatic parenchyma [2,5]. Gastric metastases from other primary malignancies are rarer than metastases to the pancreas, occurring in 0.2%-0.7% of cases [4]. The most frequent primary neoplasms are lung, breast, and melanoma, with occurrences of RCC metastases to the stomach limited to a few case reports [1,4].

## Case presentation

A 49-year-old male with a history of stage IV bilateral RCC presented with five days of epigastric and right upper quadrant abdominal pain with radiation to the mid-back. Liver-associated enzymes and lipase levels were normal. CT abdomen/pelvis showed non-specific mesenteric lymphadenopathy, normal pancreatic parenchyma, and normal caliber of pancreatic and biliary ducts.

**Figure 1:**
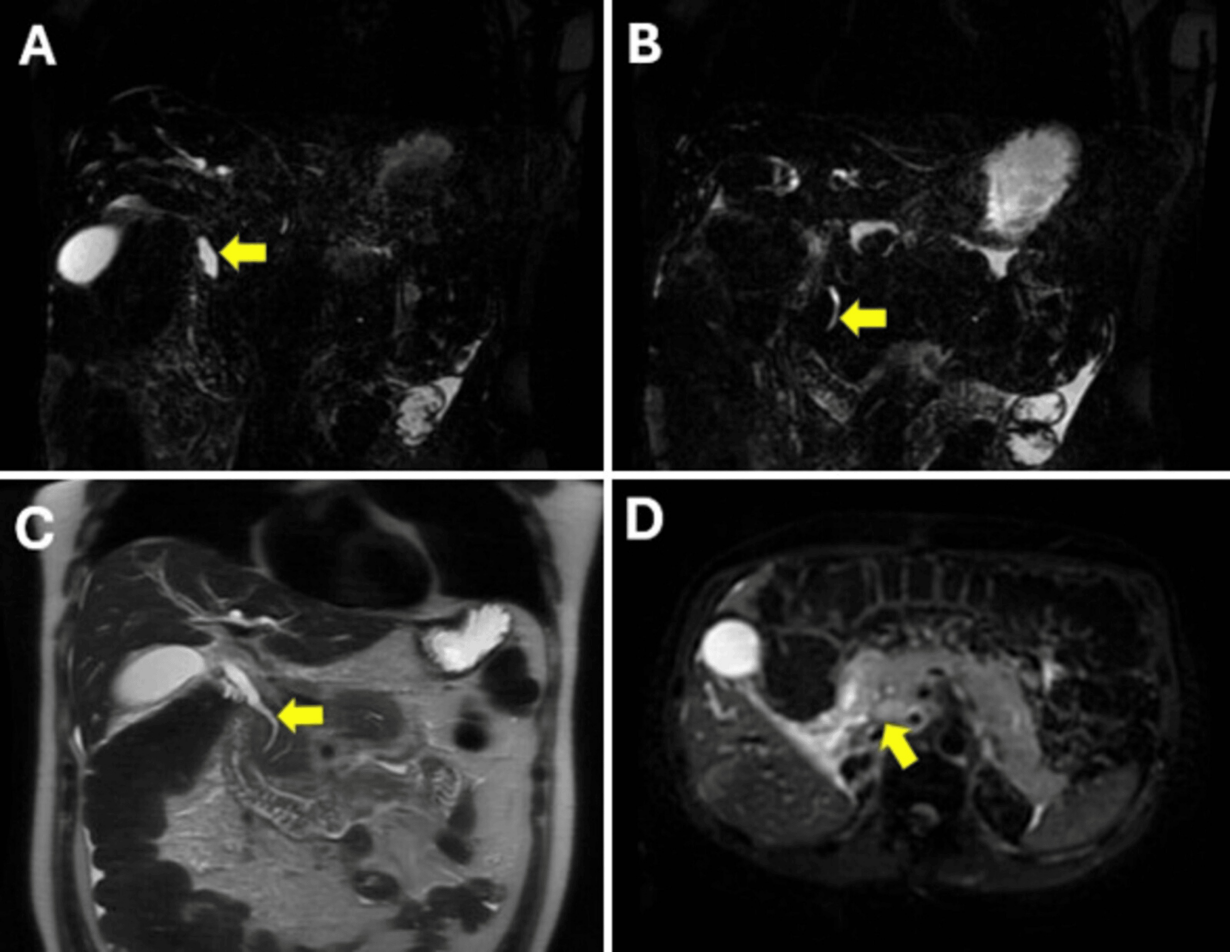
Non-contrast MRCP (A) Coronal MRCP demonstrating mid-common bile duct dilatation with (B) distal common bile duct tapering, without identifiable mass. (C) T2-weighted coronal MR image further demonstrates common bile duct dilatation with intrapancreatic narrowing, without underlying mass lesion or biliary calculus. (D) Diffusion-weighted axial (B-value=800) image demonstrating the absence of restricted diffusion at the level of the pancreatic head, consistent with the absence of visible metastatic disease at the level of the pancreas. Please note that gadolinium-enhanced MR images are not obtained due to underlying renal disease MRCP: Magnetic resonance cholangiopancreatography, MR: Magnetic resonance

One week later, he developed tachycardia and early satiety. Repeat labs were notable for elevated liver enzymes, direct bilirubinemia, and elevated lipase. Repeat CT abdomen and pelvis showed peripancreatic fat stranding and extrahepatic bile duct dilation without evidence of a focal obstructing lesion meeting Atlanta criteria for acute pancreatitis. Ultrasound and magnetic resonance cholangiopancreatography (MRCP) were negative for cholelithiasis or masses (Figures 1A-1D). The initial differential diagnosis was radiographically silent pancreatic metastases versus drug-induced pancreatitis. The only suspect drug was cabozantinib, as the patient’s dose had recently increased prior to the first admission.

**Figure 2:**
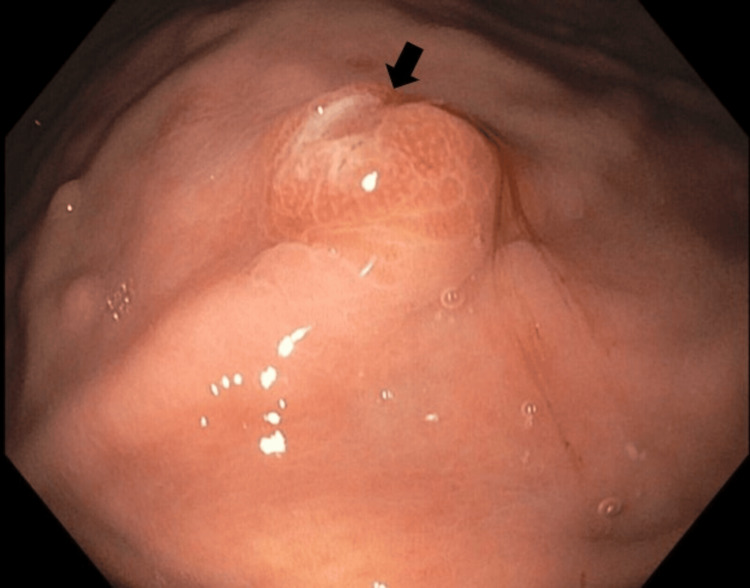
5 mm gastric nodule with central erosion

An esophagogastroduodenoscopy (EGD) was performed to rule out a peptic ulcer as another potential cause of pancreatitis. Multiple benign-appearing gastric nodules were noted. Two nodules measuring 5 mm had small erosions and were biopsied (Figure 2).

**Figure 3:**
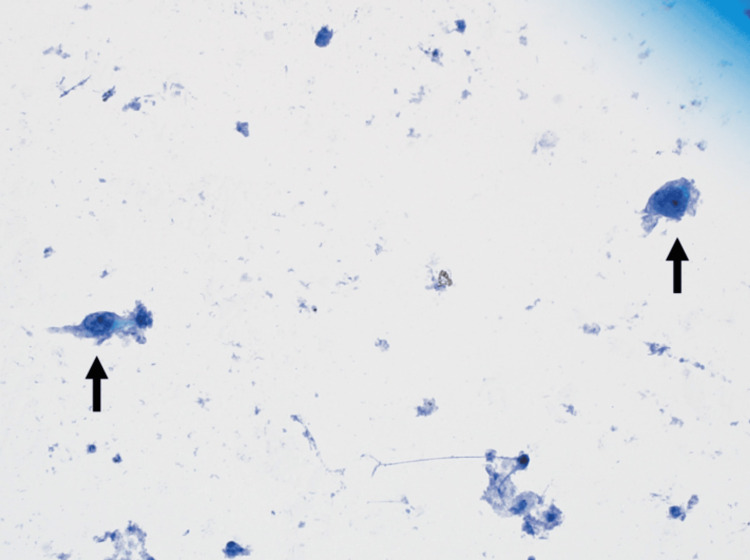
Fine needle aspiration from the head of the pancreas showing rare cells that demonstrate marked atypia with enlarged nuclei and prominent nucleoli (arrows).

**Figure 4:**
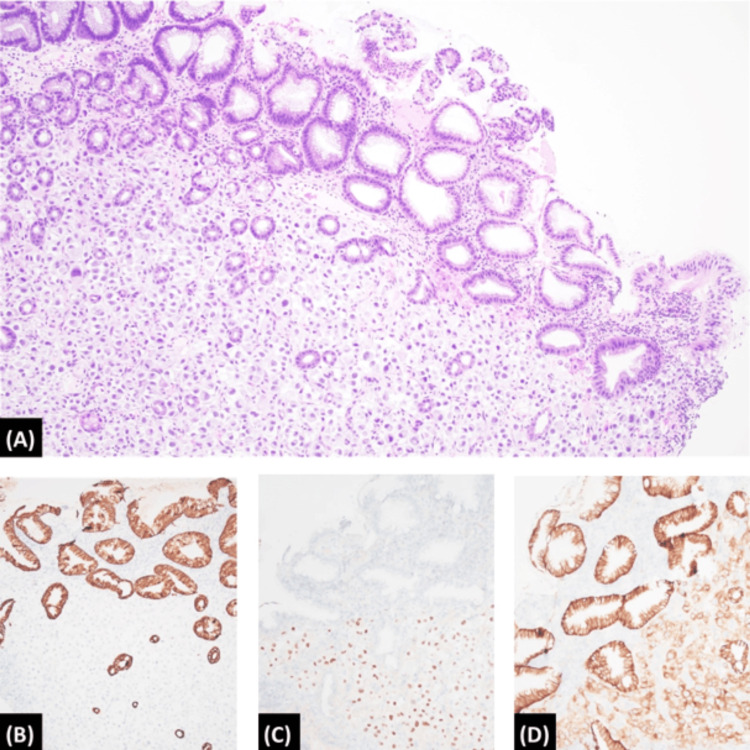
Gastric biopsy histopathology Biopsy specimen from the gastric body showed sheets of atypical, epithelioid, and rhabdoid cells with ample eosinophilic to amphophilic cytoplasm and enlarged eccentrically located nuclei, and prominent nucleoli (A; Hematoxylin and Eosin, 100x). By immunohistochemistry, the atypical cells were negative for cytokeratin AE1/AE3 (B; 100x) and positive for both PAX-8 (C; 100x) and carbonic anhydrase IX (D; 100x).

Repeat CT abdomen/pelvis showed signs of rapidly progressive disease with interval development of a fluid collection in the pancreatic tail and increasing ascites. EUS was performed when the patient did not improve after one week of standard pancreatitis management. While the EUS showed heterogeneity in the pancreas consistent with pancreatitis without any masses, it allowed for biopsies of the pancreatic head that showed atypical cells consistent with RCC metastases (Figure 3). The prior gastric biopsies also revealed metastatic RCC (Figures 4A-4D).

## Discussion

Pancreatic metastases are most frequently diagnosed in asymptomatic patients through incidental findings on imaging [3]. The patient was unusual in that he presented with a high clinical suspicion of pancreatic cancer-related pancreatitis. Multiple imaging modalities performed within two weeks of each other showed no identifiable masses even though the patient had clinically worsened rapidly. His symptoms were ultimately attributed to RCC metastases to the pancreas after EUS-guided biopsies confirmed the diagnosis.

Pancreatitis as an adverse effect of cabozantinib was considered when no focal lesions were seen, or other causes of pancreatitis were identified due to an association of tyrosine kinase inhibitors (TKIs) and the development of pancreatitis. One meta-analysis assessing the relative risk of pancreatitis associated with vascular endothelial growth factor receptor (VEGFR) TKIs, such as cabozantinib, found a relative risk of TKI pancreatitis to be 1.95 compared to 1.89 in those not on TKI therapy. Pancreatitis, however, was not expressly mentioned as an adverse event in the randomized controlled trials for cabozantinib [6]. In the clinical trial of 331 patients for its FDA approval, cabozantinib compared with controls on everolimus was associated with an increase in lipase in 2.7% (0.9% of controls) and abdominal pain in 3% (0.9% of controls). Only one patient discontinued the medication due to pancreatitis, while none of the controls discontinued it for this reason [7]. Metastatic disease was suspected in this patient due to the rarity of cabozantinib-induced pancreatitis in the current literature and the patient's clinical decompensation despite discontinuing cabozantinib.

RCC with metastases is associated with a poor five-year overall survival [8]. With its tendency to metastasize to the pancreas more frequently than many other cancers, it would be prudent to suspect pancreatic metastases whenever a patient with a history of RCC presents with vague abdominal symptoms including early satiety, pancreatitis, or laboratory findings consistent with biliary obstruction [4]. When causes such as medications, ulcers, and cholelithiasis are ruled out and symptoms fail to resolve after first-line management of pancreatitis, the metastatic disease should remain high on the differential despite the absence of mass lesions on initial imaging [3]. Early consideration of a more sensitive modality including EUS could be beneficial when suspicion is high for metastatic disease despite the absence of focal disease as well as in surgical planning if the patient has a solitary pancreatic metastasis [8,9].

Gastric lesions were also incidentally discovered on EGD which were grossly benign appearing but were found to be consistent with RCC on histopathology. Metastases to the stomach from any primary cancer are a rarer occurrence than metastases to the pancreas [4]. The available case reports describe the appearance of metastatic RCC lesions in the stomach to be distinctly protruding, polypoid with a central depression, or bleeding easily on contact with an endoscope [4]. Two of the nodules found in this case had central erosions. Obtaining histopathology on any gastrointestinal lesions, such as benign-appearing gastric polyps, may further advance the diagnosis of RCC metastases despite the rarity of gastric metastases.

## Conclusions

This report shows that metastatic RCC may demonstrate radiographically absent pancreatic metastases on CT and MRCP. This case is additionally atypical because the pancreatic metastases were highly symptomatic. Due to its tendency to metastasize to the pancreas, RCC should remain a consideration. More sensitive imaging modalities such as EUS should be implemented when patients with a history of RCC present with refractory pancreatitis, biliary obstruction, and early satiety. RCC may also manifest with gastric metastases which appear as benign nodules. The case demonstrates that in patients with nonspecific gastrointestinal symptoms that are unresponsive to initial management, direct visualization with endoscopy may be beneficial so that biopsies may be taken. The reported case shows that the manifestations of RCC in the gastrointestinal system may be highly variable.
